# On social health: history, conceptualization, and population patterning

**DOI:** 10.1080/17437199.2024.2314506

**Published:** 2024-02-13

**Authors:** David Matthew Doyle, Bruce G. Link

**Affiliations:** a Department of Medical Psychology, Amsterdam University Medical Centers, Location VUmc, Amsterdam, the Netherlands; b School of Public Policy and Department of Sociology, University of California, Riverside, CA, US

**Keywords:** Social health, social relationships, loneliness, social connection, health disparities

## Abstract

We propose a psychologically-informed concept of *social health* to join physical and mental components in a more comprehensive assessment of human health. Although there is an extensive literature on the importance of social relationships to health, a theoretical framework is needed to coalesce this work into a codified conceptualisation of social health, defined here as *adequate quantity and quality of relationships in a particular context to meet an individual’s need for meaningful human connection*. Informing this novel conceptualisation, we outline eight key propositions to guide future research and theory on social health, including five propositions focused on the conceptualisation of social health and three focused on its population patterning. The former five propositions include that social health is an outcome in its own right, that health interventions can have divergent effects on social versus physical and mental aspects of health, that social health has independent effects on quality of life, that it is a dynamic and contextual construct, and that it is embedded and encoded in the human body (and mind). The utility of the social health concept is further revealed in its significance for understanding and addressing population health concerns, such as health inequalities experienced by marginalised groups.

No more fiendish punishment could be devised, were such a thing physically possible, than that one should be turned loose in society and remain absolutely unnoticed by all the members thereof. If no one turned round when we entered, answered when we spoke, or minded what we did, but if every person we met ‘cut us dead’, and acted as if we were non-existing things, a kind of rage and impotent despair would ere long well up in us, from which the cruelest bodily tortures would be a relief; for these would make us feel that, however bad might be our plight, we had not sunk to such a depth as to be unworthy of attention at all. (James, 1890, pp. 293–294)

Perhaps the most intuitive way to imagine what constitutes a healthy life is to think about waking up in the morning and feeling capable and willing to get out of bed versus not. Everyone has had the experience of waking up either physically sick or mentally down, and the idea of getting out of bed seems both impossible and unappealing. Now imagine waking up and thinking that no one in the world cares about our presence in it. A similar sentiment would take hold, and that may be the essence of what we call ‘social health’. Here we seek to elaborate upon this intuitive understanding, proposing that *social health*, *adequate quantity and quality of relationships in a particular context to meet an individual’s need for meaningful human connection*, is an integral part of what health is. Humans evolved as a social species (Bowlby, 1973; Brewer & Caporael, 1990; Buss & Kenrick, 1998; Dunbar, 1998) and it is pointless to assert that understanding human health is possible when social dimensions are excised from our definitions of it. It is therefore surprising that some researchers and scholars have aimed to understand human health divorced from its social aspect (Wade & Halligan, 2004). We argue that social health is a critical dimension of overall health, along with mental and physical health, and that a comprehensive, tripartite conceptualisation of health (displayed in Figure 1) is needed across disciplines. Rather than portraying social relationships exclusively as predictors of other health outcomes (or as moderators of effects of other constructs, such as stress, on health), we argue that social relationships should be examined as a core component of health (i.e., a health outcome) in their own right. Adoption of the concept of social health across social and health sciences will facilitate a more holistic understanding of human potential and the central importance of social relationships to a healthy and fulfilling human life.

**Figure 1.**
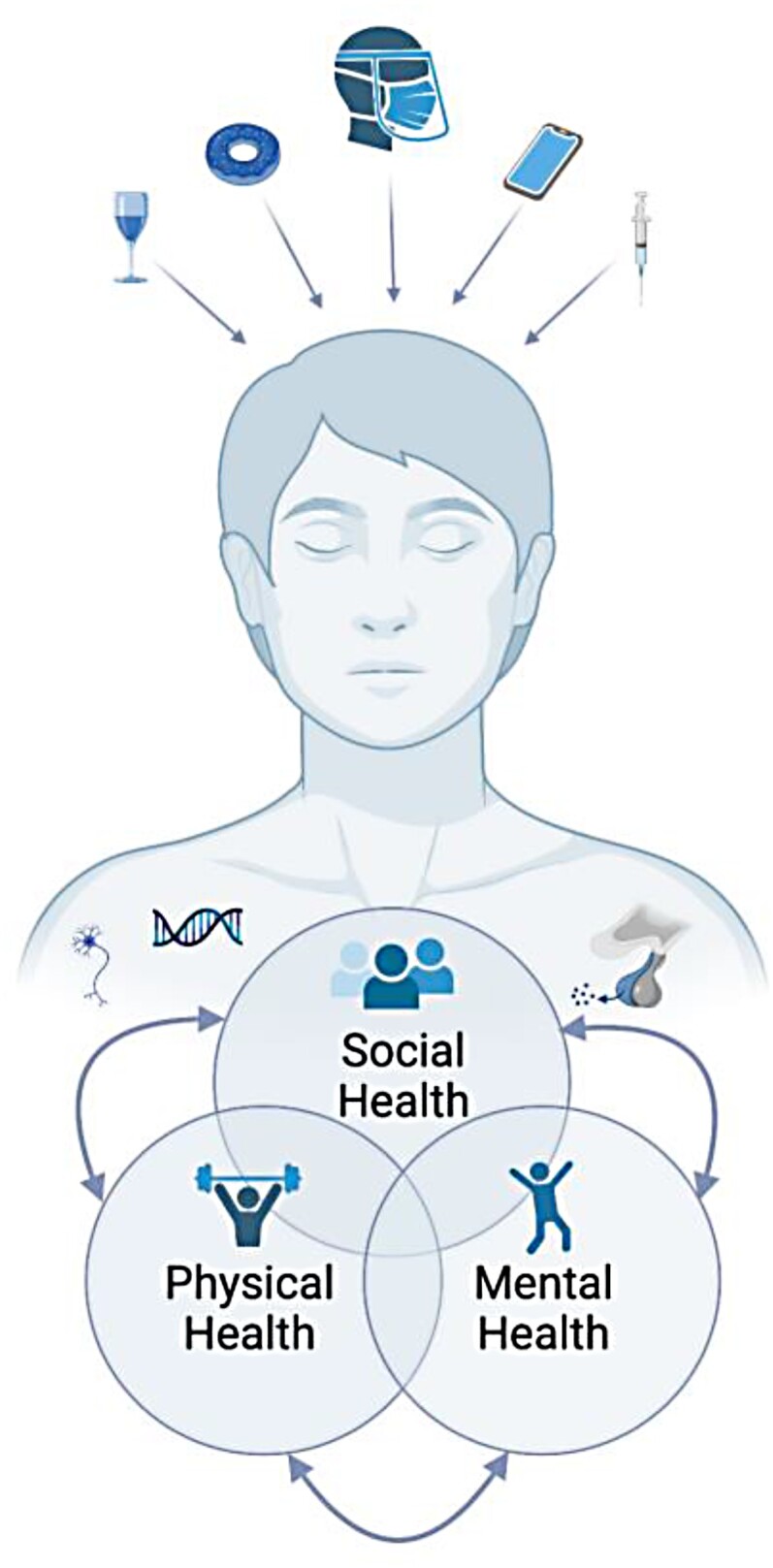
Tripartite model of health, incorporating social, physical, and mental health, situated within the human body (and mind). Figure created with BioRender.com.

What is needed to achieve this goal are evidence-based concepts and theory that can be used by social and medical scientists to further develop, test, and effectively utilize principles of social health in improving human well-being. While a strong platform for such an agenda exists in the history of medicine and in accumulating evidence from the social and health sciences, it has not up until now been brought together in a way that allows the concept of social health to realise its full potential. Our agenda is to build such a platform so that the possibility of a concept of social health might join physical and mental health in a more comprehensive assessment of human health. To achieve these aims, we begin with a brief history of the concept of health, then we propose our conceptualisation of social health and delineate some of its core features, and finally we consider the population patterning of social health. Throughout these sections, we identify eight key propositions (shown in Table 1) to help guide future work on social health, including five propositions focused on the conceptualisation of social health and three focused on its population patterning.

**Table 1.** Eight core propositions to guide future research on social health.

Proposition	
Conceptualisation of social health	
1: Social health as an outcome	Social health is an outcome in its own right, sometimes affected by mental and physical health conditions.
2: Divergent effects of interventions on forms of health	Health interventions and behaviours can have divergent effects on social, physical, and mental health (potentially risking one component while protecting another).
3: Independent effect of social health on quality of life	Social health directly shapes quality of life, even independent of mental and physical health.
4: Embodiment of social health	Social health is embodied in our biology, thus physiological mechanisms are responsible for, and responsive to, differences in social health.
5: Social health as a dynamic construct	Social health is dynamic and contextual, with decrements in some facets compensated for by fulfilment of others in a given place and time within a specific individual.
Population patterning of social health	
6: Social health as a population health issue	Level of population monitoring and investment in interventions for social health will be associated with greater well-being and reduced overall expenditure across nations.
7: Disparities in social health between groups	As with other health disparities, members of marginalised groups suffer from poorer social health on average relative to members of dominant groups.
8: Life course patterning of social health	Social health is patterned within people over time (with early life experiences especially influential in shaping adult social health outcomes).

## The concept of health through history

Our conceptualisation of social health emerges from the history of health’s conceptualisation. As we will see, social health has been integral to various conceptualizations of health across the broad sweep of human history, a circumstance that favours our reintroduction of its importance in the contemporary era. Definitions and conceptualizations of health are not static, but evolve continuously along with advances in theory and scientific knowledge as well as trends in socio-political contexts (Larson, 1999). Some of the earliest attempts to systematically understand and define health in the Western Hemisphere considered the balance of four bodily substances, or humours: blood, phlegm, black bile, and yellow bile (Sigerist, 1943). Proponents of this view, including the influential Greek philosophers Hippocrates and Galen, considered health to result from equilibrium among the humours. When disequilibrium was introduced through an imbalance of the humours, disease was thought to result. Importantly, these early medical philosophers posited that equilibrium among the humours was maintained via interactions between an individual and his or her environment, both physical and social (Breslow, 2006; Noack, 1987). This conceptualisation is also found in the seminal writings of early Eastern philosophies, including Buddhism and Taoism, which similarly view health as a state of harmony and balance (Chan et al., 2002; Wallace & Shapiro, 2006). Moreover, Eastern philosophies, such as Confucianism, emphasise the central role of well-functioning social relationships in maintaining a healthy and balanced life (Triandis, 1995; Yum, 1988). Thus, for extensive periods of time in both Eastern and Western thought, social health was considered an integral part of what health is. While Eastern philosophy and medicine to a large extent retained the principle of equilibrium (including equilibrium with the social environment; Ng et al., 2008), over time this approach has fallen out of favour in the majority of the Western hemisphere.

Scientific progress brought about many vital improvements in medicine and medical practice, but the emerging view of the body during the Scientific Revolution of the seventeenth century likened it to a machine that could be mended when component parts ceased to function ‘properly’. Disease (or ill-health), therefore, became synonymous with disruption of the system (Larson, 1999). Within the United States, the biomedical model of health dominated throughout the first three quarters of the twentieth century (Engel, 1977; Wade & Halligan, 2004). This model is situated within an understanding of health that centres on germ theory, which stipulates that disease results from the presence of specific micro-organisms capable of contaminating the body (Evans, 1976). According to the biomedical model, health can be defined as the absence of bodily disease or disability (Breslow, 1972; Guillemin & Barnard, 2015; Larson, 1999). While ascendant for a relatively brief period in the broad scope of history, this restricted biology-only model leaves little room for constructs such as mental and social health that fall outside a narrowly conceptualised physiological realm. However, as numerous scholars have observed (e.g., Johnson, 2013; Susser & Susser, 1996), the biomedical model is relatively better suited to explaining infectious diseases (the predominant causes of death and disability in the first half of the twentieth century) compared to chronic diseases (the predominant causes of death and disability in high- and middle-income countries today). As a consequence, as historical trends in patterns of disease and disability evolved, new broader approaches to health and medicine became necessary.

**Figure 2.**
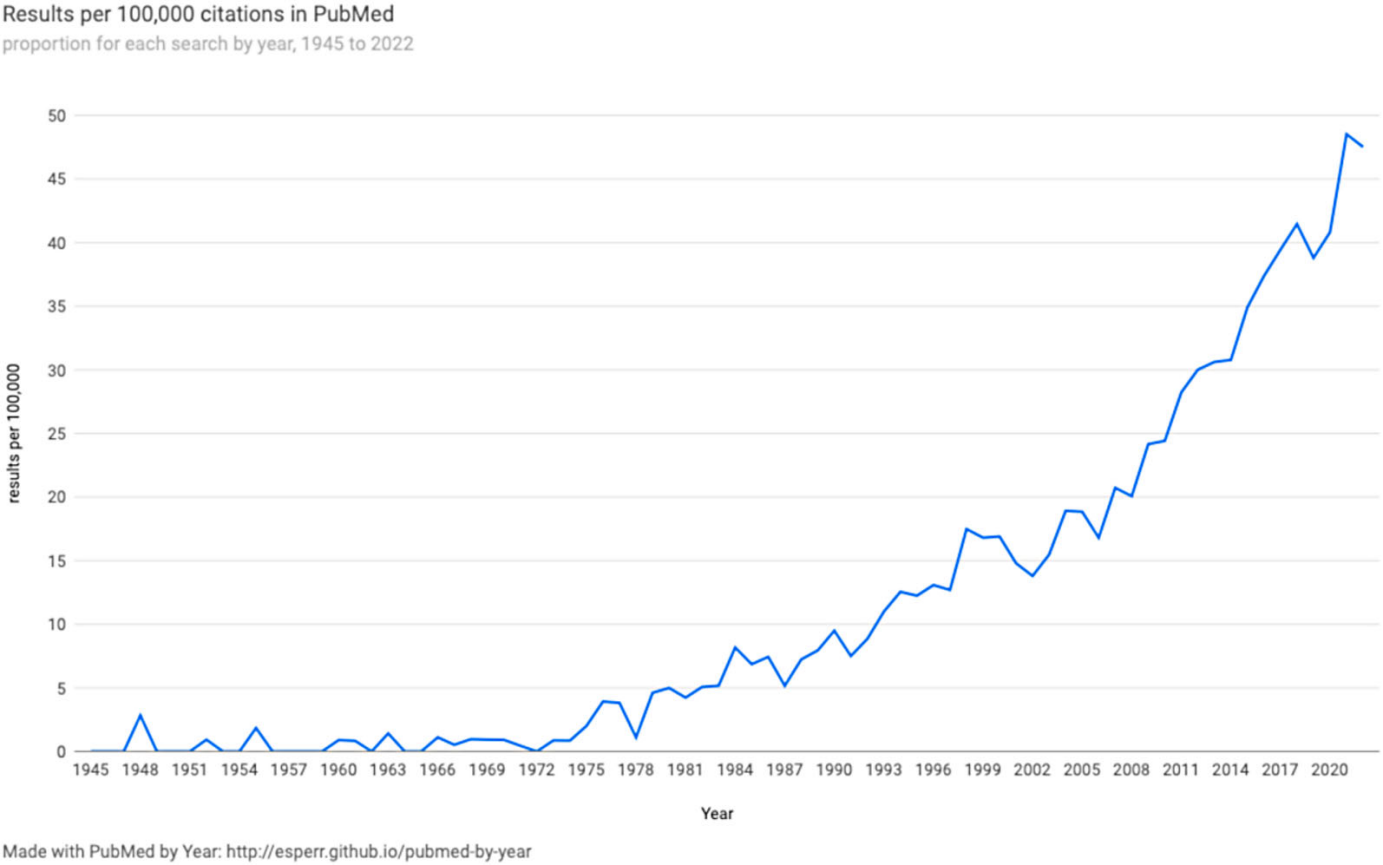
Proportion of articles in PubMed containing the term ‘social relationships’ by year from 1945 to 2022. Figure created with PubMed by Year (http://esperr.github.io/pubmed-by-year).

A watershed period for expansion of definitions of health came with the creation of the World Health Organization (WHO) and the constitution it adopted upon its founding. According to this influential global organisation, health could be defined as ‘a state of complete physical, mental and social well-being and not merely the absence of disease or infirmity’ (WHO, 1948). In proposing this definition, the WHO affirmed the necessity of understanding constructs and conditions that lie outside the physical realm. During the second half of the twentieth century, another important development came with the introduction of the biopsychosocial model of health (Engel, 1977). The biopsychosocial model was proposed to theoretically and conceptually connect social factors to biological and psychological processes (Engel, 1977; Guillemin & Barnard, 2015; Suls & Rothman, 2004). Like the WHO definition, the biopsychosocial model emerged out of a need for a more comprehensive way to understand factors that constitute health and illness, especially chronic diseases of aging (Lindau et al., 2003). Reflecting a growing recognition of the importance of social relationships to health, Figure 2 shows results of a PubMed search (Sperr, 2016) illustrating an increase in the proportion of publications in medical journals including the term ‘social relationship’ over time. Indeed, the proportion of publications including this term began increasing throughout the 1980s, perhaps driven in part by the publication of results from a key epidemiologic study linking social relationships and mortality (Berkman & Syme, 1979), with more marked increases appearing sometime in the 2000s. Despite these advances in expanding the definition of health, the biomedical model continues to heavily influence researchers and practitioners in the health sciences today (Alonso, 2004).

Scholars interested in human well-being and positive psychology (whose work reaches beyond negative states of being) have rapidly accepted social relationships as a core component of health (e.g., Keyes, 1998; Larson, 1993, 1996; Ryff & Singer, 2000). Drawing upon sociological work by theorists such as Durkheim and Marx, Keyes (1998) argued that researchers tend to emphasise private (e.g., psychological) over public (e.g., social) aspects of well-being; yet both are critical for understanding human health and, especially, thriving. This idea was elaborated and expanded upon in a theoretical and empirical review of interpersonal flourishing (Ryff & Singer, 2000), in which the authors argued that, ‘Central among the core criterial goods comprising optimal living is having quality ties to others’ (p. 31).

From this brief historical perspective, it is clear that, with the exception of a relatively recent narrow biological emphasis that is already eroding, conceptualizations of health have historically incorporated social elements. Therefore, the history of definitions of health has been to point toward social health as a core criterion. Our position is that not only researchers across the social and biological sciences, but health systems across communities and nations will benefit from the adoption of social health as a component of overall health in theory and practice. To help propel this process, we begin by outlining our conceptualisation of social health (encompassing propositions 1–5 shown in Table 1) drawn with reference to previous literature.

## Conceptualising social health

### Connecting social, physical, and mental health: a tripartite model

We argue that it is now necessary for contemporary researchers to resolutely acknowledge the fundamental position of social relationships in human health and well-being. Drawing upon the WHO’s definition of health, social health is here positioned as one of three interconnected components, along with physical and mental health. This type of overlapping model of health (displayed in Figure 1) is timely given increasing recognition of the importance of social relationships to public health (e.g., Holt-Lunstad et al., 2017) and echoes other recent proposals in the literature (e.g., Haslam et al., 2019; Karunamuni et al., 2020). Crucially, we do not assume that any one of these three components are more important or primary to health and well-being than any of the others, as some past models have suggested (Suls et al., 2010). Rather, we propose a tripartite model of health, placing social health equally alongside the other two components in health theory, research and practice. In this way, our tripartite model of health is distinct from Engel’s (1977) biopsychosocial model, which asks its users to consider each dimension in thinking about the causes, treatment, and societal management of illness, but does not treat physical, mental, and *social health* as separate distinct dimensions of health.

### Defining social health

Twenty-five years after the adoption of the WHO constitution, it was observed that relatively little effort had gone into clarifying the meaning of social health (Russell, 1973). In research with health professionals and students, Russell (1973) ultimately determined that a consensus on the definition of social health was impossible to reach at the time. Half a decade later, despite a few attempts across scientific disciplines (including psychology, psychiatry, behavioural medicine, sociology, and epidemiology), not much progress has been made on this front. Definitions of social health that do exist are infrequently cited, with researchers generally referring back to the WHO constitution to support their investigations of this construct in empirical studies. Furthermore, many important constructs that could be components of social health (e.g., loneliness, belonging, social integration) have been siloed within their respective disciplines, with highly overlapping lines of research developing largely independently from one another. For these reasons, there is a pressing need to work toward a unifying framework which will allow researchers within and across disciplines who are interested in social health to share knowledge and speak fluently. Only in this way can scholars build a comprehensive and nuanced corpus of work on social health across disciplines. If researchers are to embrace social health as a component of overall health, it is vital to agree upon a common definition to serve as a springboard for future work.

**Figure 3.**
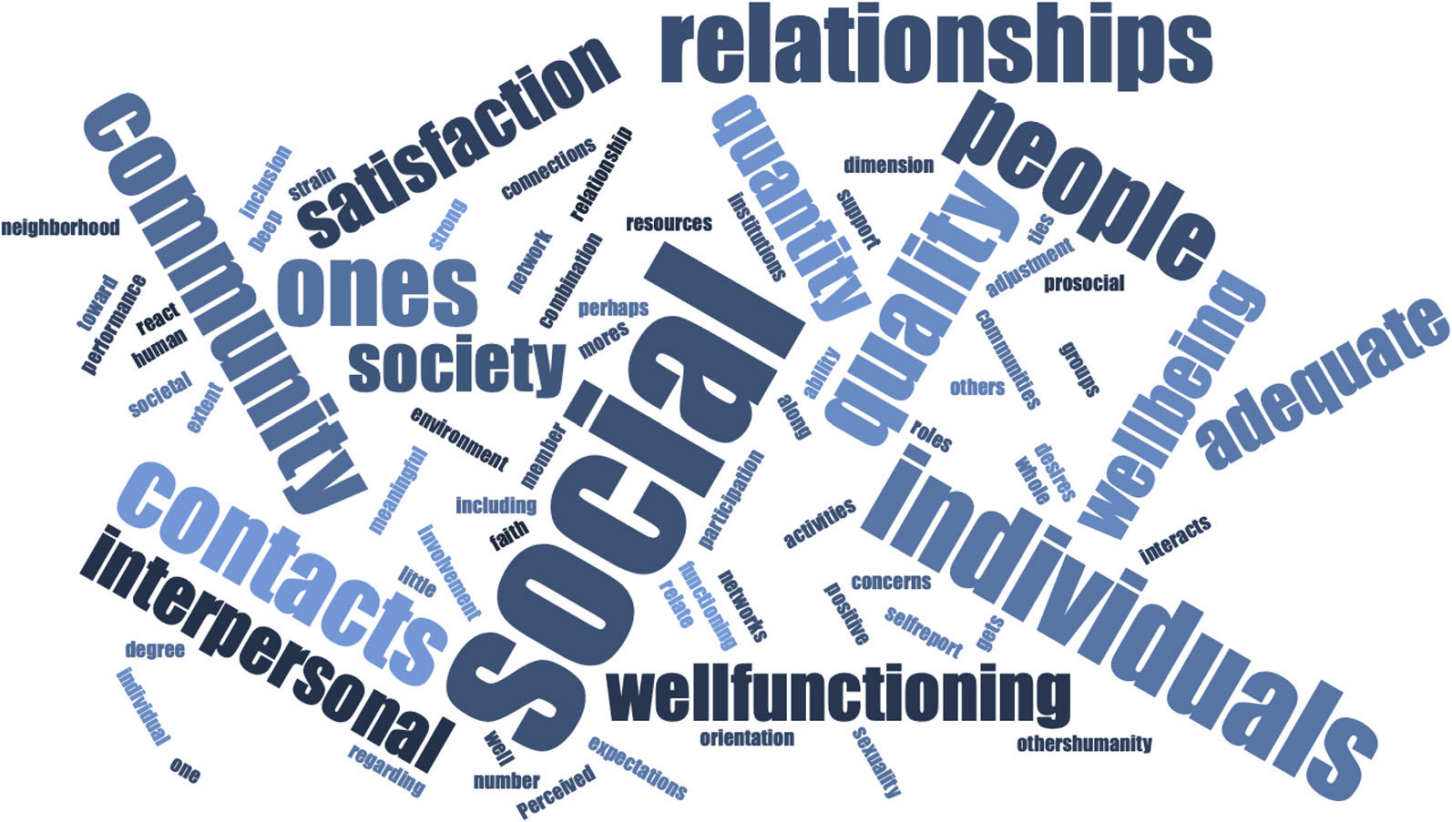
Word cloud showing terms most commonly used in definitions of social health (shown in Table 2). Figure created with Word Cloud Generator (http://www.jasondavies.com/wordcloud/).

Social health is defined here as *adequate quantity and quality of relationships in a particular context to meet an individual’s need for meaningful human connection*. This definition was developed through a careful review of relevant literature and consideration of previous definitions offered by other researchers interested in social health and well-being (shown in Table 2). These definitions vary considerably but share some important overlapping features, which we integrated to establish the current definition (see Figure 3 for a word cloud analysis using these definitions as textual data to present the most common terms). In the following sections we highlight three key elements of our novel definition drawn from this analysis and our understanding of past definitions and supporting literature.

**Table 2.** Definitions of social health/well-being in prior literature across disciplines.

Russell (1973, p. 75)	‘That dimension of an individual’s well-being that concerns how he gets along with other people, how other people react to him, and how he interacts with social institutions and societal mores’.
Renne (1974, p. 43)	‘The degree to which an individual is a functioning member of his community’.
Donald et al. (1978, p. 20)	‘The quantity and quality of an individual’s interpersonal ties and extent of involvement with the community’.
Donald and Ware (1982, p. 6)	‘The quantity and quality of social contacts and social resources’.
Larson (1993, p. 294)	‘A combination of satisfaction with relationships, performance in social roles and adjustment to one’s environment … [as well as] the number of contacts in one’s social network and satisfaction with those contacts’.
Keyes and Shapiro (2004, p. 350)	‘An individual’s self-report of the quality of his or her relationship with other people, the neighborhood, and the community’.
Castel et al. (2008, p. 738)	‘Perceived well-being regarding social activities and relationships, including the ability to relate to individuals, groups, communities, and society as a whole’.
Feeney and Collins (2014, p. 3)	‘Deep and meaningful human connections, positive interpersonal expectations, a prosocial orientation toward others, faith in others/humanity’.
Waite (2018, p. 100)	‘Adequate and well-functioning social relationships, adequate social support, little or no social strain, some social participation, social inclusion in one’s society, strong and well-functioning social networks, and, perhaps, sexuality as one desires’.

#### Social health is related to both quantity and quality of relationships

Early research on social relationships in health tended to focus exclusively on the quantitative dimension: That is, the number of social relationships to which one has access. This was partly a product of the ease with which such a dimension could be measured (e.g., asking whether one is married or single, asking how many close friends one has). Indeed, meta-analyses have confirmed that markers of social isolation, such as living alone and having infrequent social contact, are associated with mortality in addition to perceptions of isolation (Holt-Lunstad et al., 2015, 2017). However, there is growing understanding across fields that quality is just as important as quantity, if not more so (e.g., Fiori et al., 2006; Fiorillo & Sabatini, 2011). Social relationships and resultant interactions can be positive and supportive, but they can also be negative and straining, leading to impaired well-being (Brooks & Dunkel Schetter, 2011; Rook, 2015; Walen & Lachman, 2000). While the absence of high-quality relationships may be negative for social health, so too may be the presence of low-quality, taxing social ties. Of course, these qualities can also fluctuate throughout the course of a relationship, or one can hold ambivalent feelings about a given relationship at one point in time (Fingerman et al., 2004; Zoppolat et al., 2023). Importantly, because the number of truly close relationships is necessarily limited (Sutcliffe et al., 2012), the quality of these closest social ties may be particularly vital to social health – these relationships form the bedrock of most people’s experiences in the social world and consequently their well-being (Diener et al., 2000; Glenn & Weaver, 1981).

#### Like other components of health, social health is situated at the individual level

We conceptualise social health as an individual attribute that, like physical and mental health, is strongly influenced by the external social environment. As others have noted (e.g., Donald et al., 1978; Russell, 1973), there have been attempts to conceptualise social health exclusively as a property of societies. While this approach is not incorrect, it is inconsistent with predominant views of other forms of individual health – physical and mental health are typically viewed as properties of individuals. As discussed later in this paper, societal factors certainly are responsible for patterning social health, but social health is ultimately considered here to be a property of the individual. A critique that has been levelled against social health is that it extends ‘beyond the skin’ – that is, it is not entirely bound within the individual but is also a function of the social environment (e.g., Ware et al., 1981). For example, one may suffer poorer social health as a result of the death of a close relational partner (Lehman et al., 1987; Liu et al., 2019). Research has demonstrated that humans do not think and act untethered from social relationships, but rather carry with them mental imprints of relational partners (e.g., Chen et al., 2006; Fitzsimons & Bargh, 2003; Shah, 2003). For example, subtly activating mental representations of relational partners has been shown to influence goal-directed behaviours consistent with those relationships (Fitzsimons & Bargh, 2003). According to the self-expansion model (Aron & Aron, 1986), relational partners are motivated to share resources, perspectives, and even identities. Therefore, to some extent, individual’s social partners become internalised and embedded within the self, and this may even be represented at a neural level (Beckes et al., 2013). Given such evidence, it is easy to see how one could conceptualise a change or loss of social relationships as a change in individual social health.

#### The fundamental human need for social connection is central to social health

Our definition also references the need for human connection or belonging (Baumeister & Leary, 1995; Maslow, 1943), which can be fulfilled through a variety of different types of social relationships. Belonging has been described as a fundamental human need, akin to sustenance or safety (Baumeister & Leary, 1995). A sense of belonging has been defined as, ‘the experience of personal involvement in a system or environment so that persons feel themselves to be an integral part of that system or environment’ (Hagerty et al., 1992, p. 173). Although shaped by the social, cultural and environmental context, a sense of belonging is ultimately a subjective experience (Allen et al., 2021). What constitutes meaningful social connection in a given context may vary from person to person, as well as within people over time. Importantly, individuals must balance the need for social connection with the need for autonomy and distinctiveness (Brewer, 1991; Deci & Ryan, 2014); however, these two motivations are not as opposing as they may appear at first glance. That is, relationships marked by autonomy support, both received and provided, are perceived as high quality and uniquely related to the fulfilment of belonginess needs (Deci & Ryan, 2014). Of course, all forms of need fulfilment contribute to health in various ways (e.g., proper sustenance is necessary to maintain physical health, but also to prevent cognitive and psychological problems and decline; Gundersen & Ziliak, 2015), but it is perhaps almost self-evident to point out that social health is ultimately driven by a need for belonging. Therefore, our definition of social health highlights the fundamental need for meaningful human connection.

Drawing upon this novel definition, we now turn to the first five propositions guiding our conceptualisation of social health (shown in Table 1), beginning with an attempt to completely flip past research linking social relationships to health on its head by framing social health as an outcome in its own right.

### Social health as an outcome

#### Proposition 1: social health is an outcome in its own right, sometimes affected by mental and physical health conditions

##### Current conceptualisation and practice have placed social relationships as predictors of physical and mental health

Dominant current theoretical models linking social relationships and health (e.g., Berkman et al., 2000; Hawkley & Cacioppo, 2010; Pietromonaco & Collins, 2017; Robles et al., 2014) make strong arguments for the importance of social relationships in shaping human health and well-being. However, in each of these models, social health is positioned as a predictor of mental and physical health outcomes, overlooking its importance as a dependent variable. The starkest example of this consistent conceptual placement is from work on social support (the most widely investigated construct related to social relationships and health), which has been extensively discussed in terms of a main effect versus buffering model (i.e., positioning social factors as either an independent variable or moderator of the effects of stress on health; Cohen & Wills, 1985). Instead, we argue that social health can, and *should*, be considered a health outcome in its own right.

This conceptual positioning of social health as an outcome has important implications for how research in the social and health sciences is conducted and reported. An example germane to this argument comes from the experience of one of the authors when submitting a manuscript to a leading academic journal in sociomedical sciences. The manuscript was desk-rejected, with the editor writing,

We have to prioritise papers which contribute substantially to one of the major health social sciences … We are therefore not forwarding your manuscript for further consideration, as we feel it lacks a direct health outcome. The outcome in the paper is ‘social relations’.

We suspect that such experiences are not unique to us and our colleagues. It is often difficult for editors and reviewers to see the health implications of research on social relationships unless it is spelled out in clear terms (or a physical or mental health outcome is included). For example, a large proportion of papers that focus on social health (or social relationships) as the outcome frame the introductory section around the link between social relationships and physical health/mortality, frequently citing the oft-quoted statistic that loneliness is as lethal as smoking 15 cigarettes per day (Holt-Lunstad et al., 2010). Such justifications undermine the importance of social health in its own right, that is, not as a predictor of other health-related outcomes, but as a health outcome in itself.

##### Evidence that physical and mental health conditions predict social health

In fact, there may be situations in which physical and mental illnesses, rather than being outcomes, act as *predictors* of social health. A conceptual review (Henry et al., 2016) of several major psychiatric, developmental, and neurodegenerative conditions (e.g., autism, schizophrenia, Alzheimer’s disease) demonstrated that each involve decrements in social functioning as key outcomes, with implications for patients’ social relationships. Another systematic review (Daniel et al., 2009) of the literature on social consequences of stroke found consistent evidence for negative effects on family relationships, including instigation of marital problems and separation/divorce and increased parent–child conflict, as well as decreased social activities. In a classic example, major depressive disorder also tends to strain social relationships over time (e.g., Davila et al., 1997; Whisman & Uebelacker, 2009). Interestingly, proinflammatory cytokines, which have been shown to be hyperactivated in depressive disorders (e.g., Jaremka et al., 2013; Kiecolt-Glaser et al., 1987), have further been causally linked to increased social withdrawal (i.e., ‘sickness behaviors’) in non-human animals (Dantzer et al., 2008) as well as humans (e.g., Doyle & Molix, 2014c; Eisenberger et al., 2010; Inagaki et al., 2012), suggesting a potential biological mechanism. In each of these cases, social health is most appropriately considered as an outcome in health research and practice. Considering social health as an outcome, alongside physical and mental health, leads to important implications for medical practice in terms of how to best protect each of these three elements of health.

### Divergent effects on three components of health

#### Proposition 2: health interventions and behaviours can have divergent effects on social, physical, and mental health (potentially risking one component while protecting another)

Perhaps no other event in modern history has laid bare the importance of understanding social health as an outcome quite like the COVID-19 pandemic. As a result of the aggressive spread of this communicable virus, governments and health authorities across the world have needed to balance measures aimed at protecting physical health with potential consequences for mental and social health. While some nations and states have attended to all three forms of health more explicitly than others, there have been serious concerns about unintended consequences for social and mental health on a global scale (e.g., Aleman & Sommer, 2020; Bzdok & Dunbar, 2020; Galea et al., 2020; Holt-Lunstad, 2021). One of the most aggressive measures, full-scale national lockdown, has the potential undesired effect of creating social isolation, particularly for the most vulnerable groups in society (Campbell, 2020; Gauthier et al., 2021). Even less aggressive measures, including physical distancing (sometimes referred to as ‘social distancing’), may strain social health (e.g., Ford, 2020; Heid et al., 2021; Heidinger & Richter, 2020; Kovacs et al., 2021; Krendl & Perry, 2021; Lee et al., 2020; van Tilburg et al., 2020). In this case, the policies of lockdown and physical distancing, intended to protect physical health, may risk social health, requiring careful decision-making and risk mitigation efforts.

Importantly, what the example of approaches to COVID-19 control suggests is that while social, mental, and physical health are often intertwined, there are situations in which effects of interventions and behaviours on each of the three components may be separable (for a summary of other such examples, see Table 3), and understanding potentially divergent effects may be crucial to protecting overall health and well-being. Another particularly apt example of divergent effects that is directly related to healthcare is the prescription of gender-affirming hormone therapy for transgender people. Gender-affirming hormone therapy is used to medically align a patient’s body with their gender identity. While most evidence compellingly illustrates that gender-affirming hormone therapy protects mental health for transgender people (Baker et al., 2021; Doyle et al., 2023), there are potential physical health risks that sometimes require mitigation (e.g., increased risk of deep vein thrombosis; Safer & Tangpricha, 2019). Moreover, there is some limited evidence that gender-affirming hormone therapy may improve interpersonal functioning, particularly for those on masculinising hormone therapies (Doyle et al., 2023).

**Table 3.** Example of divergent effects of interventions and behaviors on three components of health.

Health interventions	Social health	Mental health	Physical health
Lockdown/social distancing	Risk	Potential risk	Protective
Gender-affirming hormone therapy	Potential protective	Protective	Potential risk
Coping through unhealthy behaviours (e.g., comfort food)	Potential risk	Protective	Risk
Social alcohol consumption	Protective	Potential risk	Risk
Social media usage	Potential protective	Potential risk	Potential risk

In terms of health behaviours, coping with stress through unhealthy behaviours (e.g., eating ‘comfort’ foods, smoking, binge-watching media) may also have divergent effects on social, mental, and physical health. While such coping strategies may buffer the deleterious effects of stress on mental health (at least for some populations; Jackson et al., 2010; Mezuk et al., 2010), they are certain to risk physical health over time. However, effects of these coping strategies on social health are likely to be mixed, with the possibility of positive outcomes due to the reduction of stress-related negative affect (which could protect relationships), but also negative effects due to the isolating nature of some of these activities (e.g., binge-watching media in privacy). Social drinking is a normative behaviour across many societies, which may maintain or protect social health through active engagement with social networks and peers (Arpin et al., 2015; Seaman & Ikegwuonu, 2010). However, this behaviour certainly carries physical health risks and may have mixed effects on mental health depending upon tendency toward addiction. Although findings for the health effects of social media usage are frequently inconsistent, there is reason to suspect that for general users, social media may protect social health, allowing people to connect with others more readily, building social support and social capital (Liu et al., 2016, 2018a). However, evidence for effects on mental health are more ambiguous, with some hints toward deleterious effects (Meier & Reinecke, 2021; Valkenburg et al., 2022), and the sedentary nature of social media usage may be linked to physical health risk (LeBlanc et al., 2017). Therefore, parental efforts to ban social media may protect children’s physical and mental health to some extent, but could potentially risk social health if usage is not excessive. Only by considering social health as an outcome in its own right (alongside mental and physical health) can researchers understand the full effects of health interventions and behaviours (e.g., physical distancing in response to COVID-19). Thorough consideration of each dimension could help guide health policy formation in order to avoid unintended consequences of enacting or prescribing such interventions (e.g., the global rise in loneliness documented after COVID-19; Ernst et al., 2022), which ultimately have important consequences for quality of life.

### Social health shapes quality of life

#### Proposition 3: social health directly shapes quality of life, even independent of mental and physical health

As with physical and mental health, social health (independent of these other factors) is tied to quality of life – a cornerstone of a ‘healthy’ life. Health-related quality of life, sometimes used synonymously with functional or health status, refers to a patient’s general functioning and well-being (Guyatt et al., 1993). Similar to chronic physical and mental illnesses, there is little doubt that poor social health impairs quality of life (Lu et al., 2020; National Academy of Sciences, Engineering, and Medicine, 2020). Among other consequences, poor social health leads to functional limitations and decline (e.g., Buchman et al., 2010; Perissinotto et al., 2012), increases sleep disturbance (Griffin et al., 2020), decreases cognitive ability (Boss et al., 2015), and impairs emotion regulation (e.g., van Roekel et al., 2014; Vanhalst et al., 2018). Research suggests that the damaging effects of social isolation on health-related quality of life are of a magnitude that is clinically significant and independent of physical comorbidities and depression as well as various demographic factors (Hawton et al., 2011).

One intriguing example of direct effects of social health on quality of life comes from a recently classified form of extreme social withdrawal, labelled ‘hikikomori’, that was first identified in Japan (Kato et al., 2018). This condition typically manifests among otherwise physically healthy young adults who withdraw into their own homes, rarely leaving or interacting in-person with others. One study (Chan & Lo, 2014) demonstrated that the degree of social withdrawal itself was predictive of the level of impairment in health-related quality of life. Overall, social isolation is so detrimental to quality of life (Luigi et al., 2020) that the United Nations (UN) and other international organisations have called for the abolition or extreme restriction of the use of solitary confinement in prisons, viewing the practice as an affront to human rights and a form of torture (Cloud et al., 2015; Shalev, 2009).

Being in extremely poor social health can be so detrimental to quality of life that it can even lead to the preference for ending one’s own life in certain circumstances. Over a century ago, Durkheim (1963) conducted seminal research on this topic, showing that levels of social integration, or close involvement with others, influence suicide rates. He classified this phenomenon as *anomic* suicide because he posited that it resulted from feelings of alienation and disconnection from society. Further research has continued to confirm that interpersonal factors, including loneliness and lack of social belonging, play an important role in suicide risk (Van Orden et al., 2010). Moreover, recent evidence suggests that ostracism, or social exclusion, can trigger suicidal thoughts in otherwise healthy people (Chen et al., 2020). Solitary confinement in prison, an extreme form of social isolation imposed by legal authority, has also been shown to increase risk of suicide (Luigi et al., 2020). It is relevant and troubling to note that mortality from suicide and unintentional overdose (sometimes referred to as ‘deaths of despair;’ Case & Deaton, 2020) has been increasing at staggering rates in recent years in the United States and other high-income countries (Bohnert & Ilgen, 2019), perhaps driven in part by loneliness and social isolation (Jeste et al., 2020). Notably, independent effects of loneliness on suicidality have been found above and beyond any indirect pathways through mental health disorders, including depression (Macalli et al., 2022; Stickley & Koyanagi, 2016). Overall, some people in extremely poor social health may actually consider life no longer worth living, and recognition of social health by those in the social and health sciences will help in understanding when and why this tragic situation might occur. However, optimal levels of social connection and types of social relationships necessary for good social health may shift from person to person and across various contexts.

### Social health as a dynamic construct

#### Proposition 4: social health is dynamic and contextual, with decrements in some facets compensated for by fulfilment of others in a given place and time within a specific individual

Social health can be conceptualised as a dynamic construct, with optimal levels that shift in response to present and anticipated future circumstances (Lee et al., 2021; Quadt et al., 2020). The same amount of social contact may become more or less adequate given other circumstances in a person’s life, such as changing city or country of residence (Oishi, 2010; Watt & Badger, 2009). Furthermore, the relative importance of various forms of relationships to social health may shift given changes in one another. For example, recent work shows that single people psychologically attune themselves to their friendships to a greater extent than people in romantic relationships, investing more time and effort in these friendships in order to fulfil the need for belonging (Fisher et al., 2021). Other evidence suggests that bereaved spouses may increase social engagement, for example with children or community groups, following the death of their partner (e.g., Burton et al., 2006; Utz et al., 2002). In fact, one study examining large-scale data related to communication on social-networking sites (Hobbs & Burke, 2017) demonstrated that in the aftermath of the death of a friend, interactions among the remaining social network immediately increased and remained elevated for years after, making up for the decrease in interactions resulting from the absence of the deceased friend. Moreover, these increased interactions immediately after the death were more pronounced among close friends compared to acquaintances, and there was no change in interactions with strangers. This study provides strong evidence for an adaptive and naturally occurring compensation process in the domain of social health.

Past research has demonstrated somewhat inconsistent findings regarding the benefits of sheer quantity of social relationships (e.g., Falci & McNeely, 2009; Kim & Lee, 2011; Liu et al., 2018b; Stokes, 1983). Above a certain point, additional relationships may not bolster health and well-being, and the costs of maintaining these relationships may outweigh any potential benefits (Pescosolido & Levy, 2002). Thus, there may be an inverted u-shaped association between quantity of social relationships and health outcomes (Liu et al., 2018b). Work drawing upon the social and evolutionary function of relationships (informed by research in non-human primates as well as humans) suggests that an average social network size is approximately 150, with humans relying on concentric social circles ever-closer in intimacy (Sutcliffe et al., 2012). According to this account, for most humans, the first two circles encompass core support networks, with these 15 or so people most helping the individual navigate the social world.

Tensions and trade-offs between network size, closeness, and quality highlight the importance of considering social health more broadly, as any one operationalisation may overlook intricacies of social health that can appear on balance across the multiple domains. Having just a few very close relationships of good quality with friends and family may equate to better social health than having mostly just an abundance of weaker ties of mixed or poor quality (Fiori et al., 2006; Fiorillo & Sabatini, 2011). However, it is important to consider that various individual differences may play a role. For example, past work has demonstrated that women focus more on dyadic interpersonal relationships while men focus more on collective social groups (Gabriel & Gardner, 1999). Personality characteristics such as extraversion and agreeableness, among others, may also pattern the types of relationships people seek out as well as the satisfaction and support that these types of relationships provide (e.g., Pollet et al., 2011; Swickert et al., 2002; Tov et al., 2016). It is crucial to consider that the building blocks of optimal social health may differ somewhat from person to person. Therefore, accurate and meaningful understanding of social health requires careful consideration of the individual and their own specific circumstances. Individual differences such as sex, culture, and personality traits will influence how various components reflect social health differentially for one person compared to another. In addition, the relation between these components and social health will vary within people depending upon circumstances and environments. Crucially, such a system requires intrinsic mechanisms (e.g., detectors, effectors) embedded in the human body for internal regulation (Lee et al., 2021).

### The human body (and mind) as the locus of (social) health

#### Proposition 5: social health is embodied in our biology, thus physiological mechanisms are responsible for, and responsive to, differences in social health

Under the tripartite model of health, the human body (and mind) is host to each of the three components (physical, mental, social; see Figure 1). Dualism, as opposed to monism, is the belief that the mind (or spirit) is ‘immaterial’ and has no corporeal form; that is, it is distinct from one’s physical body. Although few researchers in the social or health sciences today endorse the notion of a discrete separation between mind and body (Damasio, 1994), this perspective pervaded Western thought for much of the past four centuries. Our model proposes that all elements of health are encoded in the human body (and mind), including social elements. This is not as radical a proposal as it may seem upon first glance, and in fact, decades of research have demonstrated that social stimuli are processed and experienced via biological systems and that these same systems, in turn, shape social emotions, cognitions, and behaviours (Cacioppo et al., 2000, 2015; Cozolino, 2014; Feldman, 2017; Hofmann et al., 2014; Schulkin, 2011). The social nature of human biological systems is so evolutionarily ingrained that researchers have proposed the idea of a *social brain* (Atzil et al., 2018; Dunbar, 1998; Insel & Fernald, 2004; Porcelli et al., 2019).

There is now mounting evidence for core biological systems involved in social processes and social relationships. At the neuroendocrine level, the oxytocinergic system is critical in social bonding and other social cognitive processes in humans as well as non-human animals (Bartz et al., 2011; Carter, 1998; Insel, 2010). Oxytocin, along with vasopressin, prolactin, and endogenous opioids, are vital in establishing the first social bond between parent and child (Carter, 1998; Feldman & Bakermans-Kranenburg, 2017). Consistent with attachment theory, the oxytocinergic system is later stimulated in warm touch and affection between adult romantic partners (Holt-Lunstad et al., 2008) and tracks closely to relational commitment and stability (Schneiderman et al., 2012; Van Anders et al., 2011). Furthermore, this system seems to be involved in shaping the ability of social support to soothe the biological stress response (e.g., buffering the hypothalamic–pituitary–adrenal axis; Hostinar et al., 2014). Beyond its role in close relationships, the oxytocinergic system also appears to pattern intergroup behaviour and processes to some extent (De Dreu, 2012). Oxytocin may even be central to the experience of social isolation and loneliness (Carter, 2014). Functioning of the oxytocinergic system is patterned in part by genetic variations (e.g., in the oxytocin-receptor gene), which are further subject to epigenetic regulation by social experiences (Kumsta et al., 2013), all of which influences social functioning (Maud et al., 2018). It is worth noting that there is currently ongoing controversy over methods for the measurement and manipulation of oxytocin in humans (for discussion, see, e.g., MacLean et al., 2019; Quintana et al., 2018), which may limit some of the cohesiveness of research in this area and leaves room for further elaboration of the role of the oxytocinergic system in human social relationships.

At the neural level, some researchers have proposed that overlapping brain regions are responsible for processing both physical and social pain (Eisenberger et al., 2003). Similarly, experiencing pain oneself may share activation of neural regions with empathic responses to others’ pain (Singer et al., 2004). While the exact interpretation of this regional overlap is currently being debated (e.g., Lamm & Majdandžić, 2015; Wager et al., 2016), there is evidence that social relationships are deeply embedded in neural systems. Notably, the amygdala, dorsal anterior cingulate cortex, periaqueductal gray, and anterior insula appear to be most involved in social threats and disconnection, while the ventromedial prefrontal cortex, ventral striatum, and septal area appear to be most involved in social support and connection (Eisenberger & Cole, 2012). In particular, converging lines of research suggest a central role for the amygdala network in anchoring social functioning (Bickart et al., 2014). Importantly, all of these neural regions interact with other biological systems (neuroendocrine, autonomic, and immune) in the regulation of and response to social stimuli in humans.

Another line of evidence for the embodiment of social relationships comes from work on biobehavioral synchrony between relational partners (Feldman, 2017; Timmons et al., 2015). At the behavioural level, some research demonstrates that people engage in nonconscious mimicry of social interaction partners, which can facilitate liking (Chartrand & Bargh, 1999). Behavioural synchrony may be once again shaped by early parent–child interactions (Feldman, 2007) and carry over into adult close relationships (Ulmer-Yaniv et al., 2016). Beyond the behavioural level, research with romantic couples demonstrates a degree of synchrony across biomarkers, such as oxytocin (e.g., Ulmer-Yaniv et al., 2016), heart rate (e.g., Helm et al., 2012), and respiratory sinus arrhythmia (e.g., Helm et al., 2014). These linkages once more reflect the extent to which social relationships are intertwined with individual (and dyadic) biology. Increasingly, scientists are beginning to recognise that human biology is incredibly sensitive (in both a reactive and anticipatory fashion) not only to the external (e.g., Schulkin, 2011) but also to the internal social milieu (e.g., Picard & Sandi, 2021). Critics have indeed pointed out that despite its rejection of dualism, the biopsychosocial model, as proposed by Engel and advocated by others, falls prey to dualism by treating biology as concretely separate from psychosocial factors (Ghaemi, 2011). Instead, we suggest a tripartite model, in which human health is viewed as a product of each of these three components, situated within the human body (and mind). Having established this conceptualisation of social health, we now turn our attention to its implications for the health of the public (corresponding to propositions 6–8 shown in Table 1).

## Population patterning of social health

### Enabling social health in populations

#### Proposition 6: level of population monitoring and investment in interventions for social health will be associated with greater well-being and reduced overall expenditure across nations

Poor social health has enormous costs to society, both social as well as economic (Mihalopoulos et al., 2020). For example, one study of the cost of illness (Fulton & Jupp, 2015) estimated that chronic loneliness costs an average of £11,725 (equivalent to approximately $16,000) per person over a medium term (i.e., 15 years), including a variety of both medical (e.g., in-patient costs) and non-medical costs (e.g., costs of residential care). Similarly, estimates from a nationally representative sample in the Netherlands (Meisters et al., 2021) suggest that loneliness cost €3.46 billion (equivalent to approximately $4.2 billion) in healthcare expenditure in 2017. Generally, such estimates are conservative in that they cannot incorporate all of the various ways in which poor social health impairs life trajectories (e.g., worse educational and career prospects, lack of benefits and security related to marriage and family), yet the enormous costs to society are still readily apparent.

Societies can enable (or inhibit) social health through health systems, public policies, and social norms that promote (or hinder) cultures and environments allowing for the flourishing of healthy social relationships (Hinchliffe et al., 2018). One example of a holistic approach that has potential to enable social health and increase longevity comes from research on ‘blue zones’ (Buettner & Skemp, 2016; Poulain et al., 2013). Blue zones are geographic regions around the world with the highest proportion of centenarians (e.g., the Nicoya peninsula in Costa Rica, the Okinawa islands in Japan). It has been noted that these regions share health-promoting features that incorporate factors related to physical, mental, and social health. For example, in addition to culturally-embedded stress reduction techniques (e.g., prayers, napping) and healthy diets (e.g., plant-based diets, moderate alcohol consumption), these regions are characterised by a strong sense of belonging and close social ties (e.g., frequent social interactions, faith-based community participation; Buettner & Skemp, 2016). In Okinawa, people use the phrase *yui-maru* to refer to a ‘spirit of mutual aid and sense of trust and reciprocity’ in the community (Shirai, 2020, p. 305). While public health and policy attempts to artificially recreate such zones in other parts of the world have been justifiably critiqued for engaging in ‘libertarian paternalism’, potentially seeking to ‘nudge’ behaviour rather than form healthy cultures and environments (Carter, 2015), it is still valuable to consider how these societies that are successful in enabling longevity tend to prioritise social alongside physical and mental well-being.

In several countries around the world, governments and communities are beginning to consider various approaches to enabling social health. For example, cohousing (originally developed in Denmark, Sweden, and the Netherlands; Sargisson, 2012), in which residents occupy private dwellings alongside communal areas and shared spaces managed by the community, may potentially benefit social health (although there is currently only limited evidence from typically low-quality studies to support causal effects on these outcomes; Warner et al., 2020). Within the United Kingdom, social prescribing has recently been developed as part of a holistic approach to healthcare that is person-centered and can help to address social determinants of health, including loneliness and social isolation (Kellezi et al., 2019). Social prescribing aims to link primary healthcare services to community and voluntary sector organisations, such as befriending groups, bereavement groups, and volunteering organisations (South et al., 2008). As with cohousing, evidence suggests beneficial effects but comes primarily from a limited number of low-quality studies (Bickerdike et al., 2017; Reinhardt et al., 2020).

Much of the evidence-base for interventions aimed at improving individual social health comes from research on trials targeting loneliness. Meta-analyses of diverse loneliness interventions among adults (Masi et al., 2011) as well as youth (Eccles & Qualter, 2021) reveal consistent and moderate average effect sizes across reviews, with high variability in effect sizes between interventions. Similarly, systematic reviews of loneliness interventions among older adults (e.g., Gardiner et al., 2018; Poscia et al., 2018) tend to support positive effects, although noting the weakness of the quality of evidence in general. Limited evidence also hints at the ability of various interventions to increase social capital (Flores et al., 2018), social support (Hogan et al., 2002), and social group identification (e.g., Steffens et al., 2021). Perhaps the longest history of intervention in social health comes from work in the domains of marital and family therapy. A recent review of the literature on various clinical interventions for couples (Bradbury & Bodenmann, 2020) concluded that most are effective in reducing relationship distress and increasing relationship satisfaction and stability, with effects sometimes enduring after couples have exited therapy. Many preventative interventions for couples also show evidence of preventing relationship distress, albeit to a weaker extent. In line with our tripartite model, couple therapy also appears to benefit relationships compromised by mental and physical health disorders (e.g., depression, cancer), leading to marked improvements in relationship satisfaction (Bradbury & Bodenmann, 2020) along with increased quality of life (Badr & Krebs, 2013).

Generally, interventions that have been tested (or evaluated through clinical trials) focus on improving social health for those already experiencing poor social health or those who are most at risk of experiencing poor social health in the future. A different approach that has not received much attention, despite some calls within the scientific literature (e.g., Holt-Lunstad, 2018), is to attempt to implement a minor shift of the social health curve at the population level rather than focusing exclusively on at-risk individuals (Rose, 1985). This is more consistent with the blue zones approach or potentially cohousing models, which have been increasing in popularity in various countries (Krokfors, 2012), but other novel population-based interventions targeting social health could be developed on a larger scale. In addition to considering interventions aimed at enabling social health, it is crucial that health systems begin to monitor social health in the population (Hashemi et al., 2016; Holt-Lunstad et al., 2017) in addition to mental and physical health. Assessments of social health should be performed in routine healthcare and measures of social health should be embedded in population-based surveys. Only through careful monitoring of population-level social health can governments and societies ensure the overall well-being of their people. As highlighted previously, enabling social health has social and economic benefits for societies (Mihalopoulos et al., 2020), but it should ultimately be motivated by the goal of ensuring human rights (Sen, 2008; Susser, 1993).

### Disparities in social health

#### Proposition 7: as with other health disparities, members of marginalised groups suffer from poorer social health on average relative to members of dominant groups

An ever more pressing reason for researchers to swiftly reconsider the notion of social health in the population (as well as how best to monitory it) is the accumulating literature suggesting that this construct may be socially patterned in vital ways. Across diverse demographic characteristics, including but not limited to race, sex, age, sexual orientation, and socioeconomic status, differences in social health are becoming increasingly apparent (Waite, 2018). A striking pattern that has caught the attention of prior scholars (e.g., Doyle & Molix, 2014b; Hatzenbuehler et al., 2013; Umberson & Montez, 2010) is that members of stigmatised or devalued groups tend to evidence poorer social health relative to members of dominant groups. That is, as with entrenched mental and physical health disparities (Adler & Rehkopf, 2008; Braveman et al., 2011; Hatzenbuehler et al., 2013), there is strong and growing evidence for disparities in social health.

For example, sexual minorities tend to experience poorer social health relative to heterosexuals, including elevated levels of loneliness and close relationship strain as well as lower levels of social capital (Doyle & Molix, 2016). Sexual minority youth experience high levels of bullying and social rejection (e.g., Olsen et al., 2014; Poteat et al., 2020), including sometimes from their own families (McGeough & Sterzing, 2018). There is also some evidence for greater instability in same-sex romantic relationships compared to different-sex relationships (Manning & Joyner, 2019). Together, these disparities in social health may help explain elevated levels of suicide risk among sexual minority youth and adults (Plöderl et al., 2014; Poštuvan et al., 2019). Racial and ethnic minorities also tend to experience poorer social health relative to Whites (Doyle et al., 2018). For example, African American men and women are less likely to marry and more likely to divorce than White men and women (Raley et al., 2015) and report lower marital quality on average (Bulanda & Brown, 2007). Racial and ethnic minorities tend to have lower social capital (Lin, 2000) and some evidence hints at smaller social networks compared to Whites (e.g., Ajrouch et al., 2001; Miyawaki, 2015). Other research indicates that racial and ethnic minorities are also at greater risk of experiencing loneliness compared to Whites, particularly in older age (e.g., Hawkley et al., 2008; Miyawaki, 2015; Tomaka et al., 2006; Victor et al., 2012).

As with other inequalities, the COVID-19 pandemic has not only brought these disparities in social health to light, but it may also have exacerbated them (Campbell, 2020; Gauthier et al., 2021). Increased burdens of structural stressors (e.g., decreased financial stability; Bui et al., 2021) may have further strained social relationships for members of devalued groups. Moreover, inequalities in morbidity and mortality from COVID-19 (Bambra et al., 2020; Van Dorn et al., 2020) led to greater disruption and loss in social networks for members of devalued groups. Taken together, the toll of the COVID-19 pandemic on social health is likely to have been more severe for members of stigmatised and devalued groups.

In chronicling these disparities in social health, it is important to stress that evidence does not support an essentialist perspective in which members of stigmatised groups are inherently less capable of maintaining healthy and satisfying social relationships. Rather, there are myriad cultural and structural factors that work to impair social health for members of devalued groups across societies. Perhaps chief among these is exposure to prejudice and discrimination. There is now growing evidence from research with a variety of stigmatised groups (including sexual and racial minorities) that exposure to prejudice and discrimination damages social health (e.g., Chen & Yang, 2014; Doyle & Molix, 2015; Murry et al., 2001; Priest et al., 2017; Trail et al., 2012), potentially through mechanisms such as impaired self-image (Doyle & Molix, 2014a) and emotion dysregulation (Doyle & Molix, 2014c). Furthermore, experimental evidence demonstrates that exposure to prejudice and discrimination causally inhibits trust (Zhang et al., 2020), impairs close relationship quality (Doyle & Molix, 2014b), increases loneliness (Doyle & Barreto, 2024), and decreases sensitivity to signals of social inclusion (Richman et al., 2016). In one study (Doyle & Molix, 2016), perceived discrimination was shown to explain much of the gap in social health between sexual minorities and heterosexuals. Relatedly, low social rank is associated with greater social anxiety (Aderka et al., 2009), potentially leading to defensive social behaviours, such as submission and avoidance, that may inadvertently harm social health (Blay et al., 2021).

Of course, as with any outcome of interest, there are protective factors and sources of resilience for social health unique to various stigmatised groups. For example, sexual minorities often form strong bonds with ‘families of choice’ because of frequent experiences of rejection from ‘families of origin’ (Frost et al., 2016). Among many racial and ethnic minority groups, it is also common to form ‘extended families’ that encompass both actual and ‘fictive’ kin, particularly to provide support in raising children (Ebaugh & Curry, 2000; Harrison et al., 1990). More broadly, there is work to suggest that in response to discrimination and rejection from members of dominant groups, members of stigmatised groups may draw closer to other in-group members as a source of support and affirmation (i.e., the rejection-identification model; Branscombe et al., 1999). However, acknowledging these sources of protection and resilience does not obviate substantial disparities in social health that appear to be patterned across a number of different devalued social identities.

Turning back to the tripartite model of health, disparities in social health between devalued and dominant groups may help explain the development and maintenance of disparities in other forms of health across the life course (i.e., physical and mental; Doyle et al., 2018). ‘Beneficial social connections’ have been deemed a key flexible resource that facilitates the recreation of health inequalities in different places and time (Phelan et al., 2010). In fact, social health may represent a pathway through which ‘fundamental causes’ (Link & Phelan, 1995), including social stigma (Hatzenbuehler et al., 2013), shape other health outcomes. Therefore, efforts to eliminate disparities in social health may have the added benefit of effectively reducing other entrenched health inequalities as well. As with physical and mental health, we have demonstrated that there are important disparities in social health that must be attended to by future researchers.

### Social health over time and across generations

#### Proposition 8: social health is patterned within people over time (with early life experiences especially influential in shaping adult social health outcomes)

Research demonstrates that social health also fluctuates throughout the individual life course (Blieszner, 2006; Ertel et al., 2009; Luong et al., 2011; Mund et al., 2020; Wrzus et al., 2013), just as physical and mental health do (Ben-Shlomo & Kuh, 2002). As with other forms of health, there is growing evidence that social health is patterned from early life through adulthood and into older age (Doyle & Cicchetti, 2017; Simpson et al., 2011; Umberson et al., 2016). Attachment theory (Bowlby, 1973) proposes that the presence of caring and responsive relational partners (serving as a ‘secure base’) allows one to explore the social environment and cope with novel and potentially threatening social stimuli in a healthy manner. Attachment theory was originally developed with reference to the parent–child relational bond (Ainsworth et al., 1978; Bowlby, 1973) and was subsequently expanded to include carry-over to adult relational partners (Hazan & Shaver, 1994). Attachment patterns are thought to form early on and persist throughout development (Fearon & Roisman, 2017; Fraley, 2002), although there is some evidence that they are capable of change from one relational partner to another (e.g., La Guardia et al., 2000). The importance of early life experiences (which shape initial attachment styles) is reflected in the fact that social health is intimately bound not only to quality of life, as discussed previously, but also to life chances. Just as with physical and mental health, social health patterns so much about a person’s life trajectory, including educational attainment (e.g., Dufur et al., 2013), career prospects (e.g., Seibert et al., 2001), residential independence (e.g., Seiffe-Krenke, 2006), and family formation (e.g., Thorsen, 2017).

In addition to patterning throughout the individual life course, there is some evidence for shifts in indicators of social health across generations (which are mirrored by shifts in predominant physical illnesses, as described previously, as well as increasing prevalence of certain mental health conditions over time, such as autism spectrum disorder; Myers et al., 2019). For example, it has been argued that social capital is decreasing on average over time in the United States (Putnam, 2000). Examples of this decline include shrinking membership in community groups and voluntary organisations, less time spent on informal visiting and socialising, and decreasing church attendance (Putnam, 1995). Other work has demonstrated a reduction in the size of average American discussion networks, including with kin and non-kin, between 1985 and 2004 (McPherson et al., 2006). A recent meta-analysis also showed evidence for a lower prioritisation of values of affiliation and community across generations in the United States (Twenge et al., 2012). On a more global scale, there has been a dramatic rise in the number of people living alone, particularly since the 1960s (Snell, 2017). This shift in living patterns corresponds to rising reports of loneliness, which have been substantial enough to lead some academics as well as mass media to label this phenomenon a ‘loneliness epidemic’ (e.g., Bergland, 2015; Holt-Lunstad, 2017; Murthy, 2017).

Despite such seemingly dramatic changes in human health and well-being over time, some of these patterns have been empirically challenged or reinterpreted through the lens of shifting social norms around individualism and independence as well as the rise of technology and social media (e.g., Bound Alberti, 2018; Hampton & Wellman, 2018; Klinenberg, 2018; Suanet & van Tilburg, 2019). Undoubtedly, technology is shaping ways in which social relationships are mediated in the current era (Bayer et al., 2020; Kross et al., 2020), which could have implications for social health on a broad scale. More than ever before, the COVID-19 pandemic has transformed people’s social lives, increasing time spent socialising via digital technology and on-line services (Ohme et al., 2020). Furthermore, some researchers are currently investigating ‘social robots’ as a solution to poor social health, particularly among older adults (Broekens et al., 2009; Pu et al., 2019). It is unclear to what extent such technological interventions will be capable of bolstering social health, with limited and generally low-quality evidence available at present (Noone et al., 2020; Pu et al., 2019). Additionally, there are ethical issues inherent in the ‘humanization’ of social robots (Giger et al., 2019). However, this remains an area of financial investment and a research priority for many societies concerned with the possibility of flagging levels of social health in the population. Shifting patterns of social health across time within individuals as well as between generations (just as with physical and mental health) necessitate a life course perspective utilising a historical lens to best capture the construct of social health.

## Conclusion

Building upon an intuitive understanding of the construct along with extensive theorising of others who came before us, we set out to explicitly conceptualise the phenomenon of social health. We situated the construct of social health within an ever-evolving and lengthy history of conceptualizations of health and well-being across societies. Building upon this foundation, we outlined eight key propositions related to social health (Table 1), specifying that it is an outcome in its own right, that health interventions can have divergent effects on social versus physical and mental aspects of health (as dramatically demonstrated by the case of social distancing), that social health has independent effects on quality of life and mortality, that it is a dynamic and contextual construct, and that it is embedded and encoded in the human body (and mind). Furthermore, we discussed costs associated with poor social health and the burden that poor social health can have on society, with our discussion of population patterning highlighting a number of ways in which demographic characteristics, including age and generational cohort, might influence social health. In particular, we sought to draw attention to pronounced disparities in social health between members of stigmatised and dominant groups that echo entrenched disparities in physical and mental health. Through strategies that enable social health across populations and cultures, such as blue zones, cohousing, and social prescribing, it may be possible to improve other domains of health (i.e., physical and mental) and ensure a state of optimal human functioning for all.

The conceptualisation we have developed will certainly be elaborated and extended, importantly perhaps by colleagues from other social science disciplines, such as sociology and anthropology. Indeed, what we see as one of the core utilities of the work so far is that it takes what we know now and organises it in a way that provides a platform that can be used to refine and extend it. As psychological and other research scientists continue to contribute evidence, that evidence can be coalesced into the framework presented here to further develop our conceptualisation of social health. We began our paper by referring to an intuition that points to the importance of social health by asking one to imagine ‘waking up and thinking that no one in the world cares about our presence in it’. While we still believe that such an intuition has value, our hope is that the conceptualisation and codification we have undertaken systematically fills in what the intuition aptly draws our attention to. We hope our efforts at conceptualisation will allow scholars across the social and health sciences to now unabashedly embrace the pursuit of social health.
